# Regeneration of Chronic Rotator Cuff Tear in a Rabbit Model: Synergetic Benefits of Human Umbilical Cord Blood-Derived Mesenchymal Stem Cells, Polydeoxyribonucleotides, and Microcurrent Therapy

**DOI:** 10.1155/2022/6496773

**Published:** 2022-03-15

**Authors:** Dong Rak Kwon, Kang Lip Kim, Yong Suk Moon

**Affiliations:** ^1^Department of Rehabilitation Medicine, Muscle Research Center, Catholic University of Daegu School of Medicine, Daegu, Republic of Korea; ^2^Department of Anatomy, Catholic University of Daegu School of Medicine, Daegu, Republic of Korea

## Abstract

**Objective:**

To investigate synergic therapeutic effects of combined injection of intralesional mesenchymal stem cells derived from human umbilical cord blood (UCB-MSCs) and polydeoxyribonucleotide (PDRN) combined with microcurrent therapy (MIC) on full thickness rotator cuff tendon tear (FTRCTT) in rabbit models.

**Methods:**

Thirty-two rabbit models were assigned to 4 different groups. FTRCTT in the supraspinatus tendon was created. After 6 weeks, 4 types of procedures (0.2 mL normal saline injection, group 1 (G1-NS); 0.2 mL SC injection, group 2 (G2-MSC); 0.2 mL SC and weekly four injections of 0.2 mL PDRN with sham MIC, group 3 (G3-MSC+PDRN+sham MIC); and 0.2 mL SC and weekly four injections of 0.2 mL PDRN with MIC for four weeks, group 4 (G4-MSC+PDRN+MIC)) were performed in FTRCTT. Gross morphologic and histological changes of proliferating cell nuclear antigen (PCNA), vascular endothelial growth factor (VEGF) and platelet endothelial cell adhesion molecule (PECAM-1) and motion analysis were performed.

**Results:**

There was a significant difference in gross morphologic changes between baseline and week 4 posttreatment in group 4 compared to the other three groups (*p* = 0.01). In groups 3 and 4, all parameters of histochemical and motion analysis have been found to be significantly greater than the ones in groups 1 and 2 (*p* < 0.05). In group 4, PCNA-, VEGF-, and PECAM-1-stained cells, as well as walking distance, were significantly greater than the ones in group 3 (*p* < 0.05).

**Conclusion:**

The treatment with UCB-MSCs and PDRN combined with MIC might be the most effective in rabbit models' traumatic FRTCTT.

## 1. Introduction

Rotator cuff tendon tear (RCTT) is one of the most leading causes of shoulder disability and pain, strongly related to advanced aging. It affects approximately 20% and 31% of population over 60 and over 70 years of age, respectively [1]. In spite of considerable improvement in current treatment strategies for RCTT, furthermore, these approaches tend to cause fibrotic tissue with far lower mechanical properties than native tendon. Moreover, it is still difficult for full rotator cuff function to be recovered. Previous studies [2, 3] showed that in the nonretracted and retracted cuff tear model of rabbit or sheep, disorganized immature fibrous tissue bound the tendon-to-bone junction with healing, although there is a difference in degree. For these reasons, treatments using biological adjuvants, which can create a favorable environment for rotator cuff tendons to be regenerated, have been in the limelight. Similar to other tissue engineering methods, tendon tissue engineering is dependent upon three aspects including cells, biomaterials, and chemically/physically affected environment [4].

Mesenchymal stem cells (MSCs) have stood out as biological adjuvants to resolve problems found in the current treatment methods. MSCs are known to potentially differentiate tenocytes, proliferate actively, and secrete paracrine factors and have immunomodulatory impacts on promotion of tendon regeneration. Human umbilical cord blood-derived MSCs (UCB-MSCs) that are generally classified as waste and discarded after deliver are isolated from umbilical cords tissue [5]. Thus, it can be obtained through noninvasive process and, accordingly, provided at low cost [6]. UCB-MSCs have been reported to have a higher proliferation rate, lower immunogenicity, and higher self-renewal potential than MSCs derived from other tissues. These characteristics may contribute to UCB-MSCs being a better therapeutic option than other adult MSCs [7]. Previous studies have demonstrated that UCB-MSCs regenerated full-thickness defects in rotator cuff tendon (full thickness RCTT (FTRCTT)) of rat and rabbit models [8–10]. Recently, there has been a study suggesting deoxyribonucleotide polymers mixed with a chain length between 50 and 2,000 bp; i.e., polydeoxyribonucleotide (PDRN) had effect on the treatment of chronic rotator cuff disease [11]. PDRN is involved in anti-inflammatory activity as well as promoting angiogenesis and collagen synthesis [12]. In a recent animal study [13, 14], the results indicate combined injection of UCB-MSCs and PDRN has more effectively improved a FTRCTT than UCB-MSCs being injected alone. It is assumed that the outcomes have been mediated by promotion of vascular endothelial growth factor (VEGF), presumably considered regeneration potential in RCTT. Among growth factors, VEGF is known to promote healing process the most by provoking new vessels to form in the regions where circulation is poor, such as a FTRCTT [11, 15]. Also, VEGF controls various biological functions of endothelial cells and enhances vasodilatory mediators to be produced, increases vascular permeability and stimulates the cells to migrate, proliferate, and form [16].

In addition, microcurrent therapy (MIC) is a subsensory stimulation treatment that involves an extremely low-level electrical current (<1 mA) [17]. Application of microcurrent stimulation has been shown to promote release of VEGF *in vitro*, in animal studies and in clinical practice [18–20]. MIC can also promote production of adenosine triphosphate, protein synthesis, and amino-acid transportation, which would reduce inflammatory activity and accelerate wound healing [21]. Based on this background, we formulated a hypothesis in animal model that coinjection of intralesional UCB-MSCs and PDRN combined with MIC would have greater effect on regeneration of a tendon tear than UCB-MSC injected alone. Hence, the effects of UCB-MSCs and PDRN accompanied with MIC on the rabbit models' supraspinatus tendon chronic RCTT regeneration have been evaluated.

## 2. Materials and Methods

### 2.1. Animal Model

**Figure 1:**
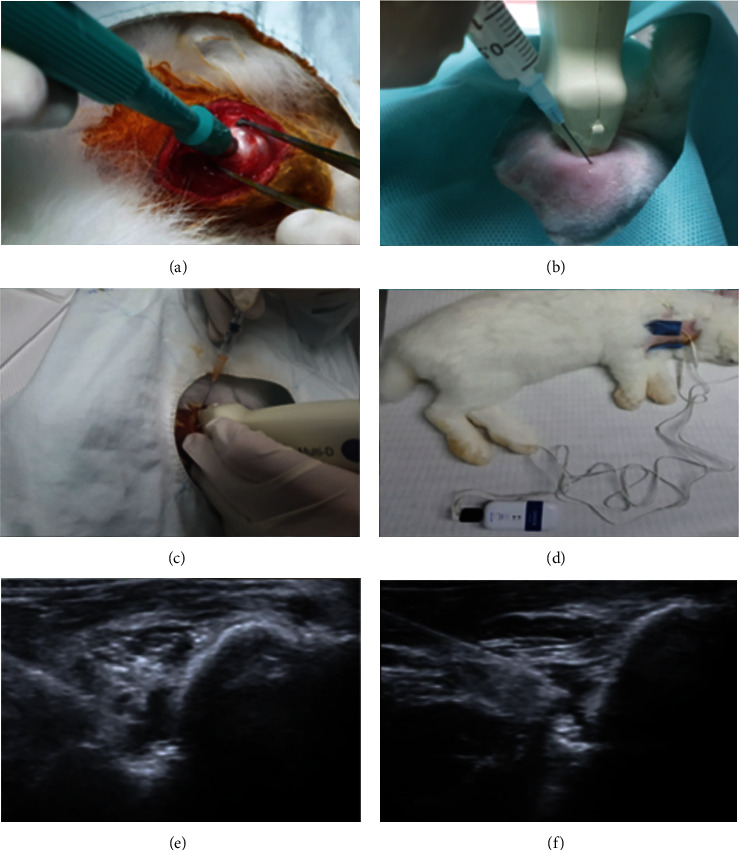
FTRCTTs were made at just proximal area to the insertion point by excision on the supraspinatus tendon (a). 0.2 mL UCB-MSCs (b) and weekly four injections of 0.2 mL PDRN (c) combined with MIC (d) for four weeks (G4-SC+PDRN+MIC) were performed into FTRCTT under US guidance (e, f). FTRCTT: full thickness rotator cuff tendon tear; UCB-MSCs: human umbilical cord blood-derived mesenchymal stem cell; PDRN: polydeoxyribonucleotides; MIC: microcurrent therapy; US: ultrasound.

According to the internationally accredited guidelines, animal experiments were implemented with approval of the Catholic University of Daegu School of Medicine Animal Care and Use Committee. Thirty-two male New Zealand White rabbits aged 12 weeks were separately placed in a detached metal crate under the condition of 45 ± 10% in relative humidity and 23 ± 2°C in temperature. Within each of the crates sized 65 × 45 × 30 cm, the subjects were enabled to have normal activities, although no other exercise was offered [9]. Also, the subjects were allowed to access to water without restriction and provided commercial rabbit food. They were induced anesthesia with isoflurane (JW Pharma, Seoul, Korea) vaporized in oxygen and delivered with a large animal cycling system. Under general anesthesia, a 5 mm FTRCTT was created on each of the right supraspinatus tendons at slightly proximal area to the muscle insertion points using a 5-mm biopsy punch (SFM, Wächtersbach, Germany) (Figure 1(a)). Wounds were promptly concealed with a silicone Penrose drainage tube in 5 mm diameter (Sewoon Medical, Cheonan, Korea) to avoid natural healing [22]. Once the FTRCTT model was prepared, the wound was sutured for subcutaneous and skin closure [9].

### 2.2. Animal Grouping and Treatment

**Figure 2:**
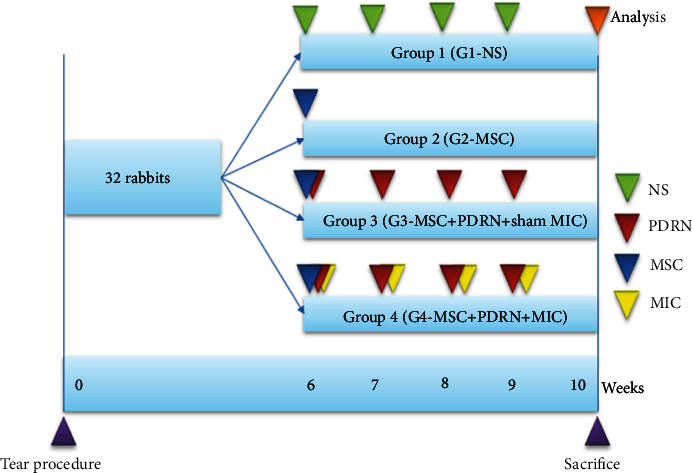
Timeline of the study. FTRCTTs were made in the right supraspinatus. Each group were treated with the following protocols: 0.2 mL normal saline injection, group 1 (G1-NS); 0.2 mL UCB-MSCs injection, group 2 (G2-MSC); 0.2 mL UCB-MSCs and weekly four injections of 0.2 mL PDRN with sham MIC, group 3 (G3-MSC+PDRN+sham MIC); and 0.2 mL UCB-MSCs and weekly four injections of 0.2 mL PDRN with MIC for four weeks, group 4 (G4-MSC+PDRN+MIC). Euthanasia of the rabbits were done at 4 weeks after treatment.

The tubes inserted in the tendons were removed in six weeks since an FTRCTT was created on each rabbit. Once FTRCTT sites were confirmed, the incisions were closed with subcutaneous and skin sutures. Thirty-two rabbits were divided into 4 groups at random (8 rabbits for each group). Four types of procedures (0.2 mL physiological saline injection, group 1 (G1-NS); 0.2 mL of UCB-MSC injection (Figure 1(b)), group 2 (G2-MSC); 0.2 mL of UCB-MSCs and 4 sessions of 0.2 mL of PDRN (Figure 1(c)) with sham MIC per week, group 3 (G3-MSC+PDRN+sham MIC); and 0.2 mL UCB-MSCs and 4 sessions of injection of 0.2 mL PDRN with MIC per week (Figure 1(d)), group 4 (G4-MSC+PDRN+MIC)) were performed in the site of the FTRCTTs for four weeks. Commercially obtained PDRN (Rejuvenex Inj., Pharma Research Product, South Korea) and UCB-MSCs (Cartistem, MEDIPOST, Korea) were used as injectates. All injections were administered under ultrasound guidance (Figures 1(e) and 1(f)). In groups 3 and 4, sham MIC or MIC has been carried out for one hour each day for 4 weeks since the first injection was administered. For MIC to be applied, an electrical pad was attached to the skin over the tear area, and a microcurrent generating device (Granthe, Cosmic Co., Korea) with an intensity of 25 *μ*A and a frequency of 8 Hz was also used. For sham MIC, an electrical pad was attached the skin surface of tear site without electrical current. Euthanasia was performed on all the rabbits 4 weeks after first injection (Figure 2) since a previous study demonstrated that the surviving number of MSCs and the number of immature fibroblast at 4 weeks after treatment were significant higher than those at 2 weeks and 6 weeks [23].

### 2.3. Preparation of MSCs

Informed consent was obtained from pregnant mothers for the use of UCB. An independent cord blood bank unit took human UCB from umbilical veins after neonate delivery. UCB-MSCs were taken according to the manufacturers' protocols of the cord blood bank [24]. A single donor provided all the UCB-MSC used in this study. The isolated MSCs were cultured 3 times prior to cryopreservation. After thawing, three more passages were performed for sufficient collection of UCB-MSC. The process for MSC was as described in previous studies [24–26]. As in a previous study [27], the expression of CD73 and CD105, but not CD14, CD34, CD45, and HLA-DR, was found in these cells. Hyaluronic acid hydrogels were prepared with hyaluronic acid (HA, Hyal 2000; LG Life Sciences, Seoul, Korea) dissolving in a minimum essential medium (a-MEM) for a composite of UCB_MSC and hyaluronic acid (HA). The optimal density of HA of 4% was found as in a previous study [28], based on cell morphological analysis, in vitro viability test, rheological characterization, and cell proliferation. The 4% HA and monolayer UCB-MSC culture were mixed completely [24].

### 2.4. Gross Morphology Examination

**Figure 3:**
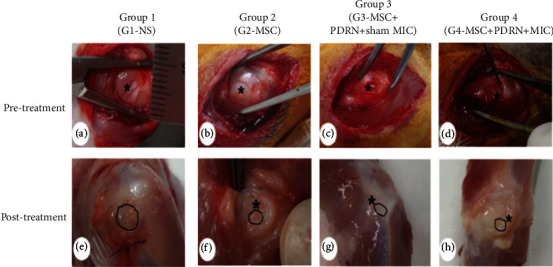
Gross morphology (a–h) of the supraspinatus tendons in each group. (a–d) Pretreatment images. FTRCTTs were found in all four groups. (e–h) Posttreatment images. FTRCTT is shown, and no changes were observed between baseline and 4 weeks posttreatment in group 1. There were significant differences between the baseline and posttreatment in group 2, group 3, and group 4.

**Figure 4:**
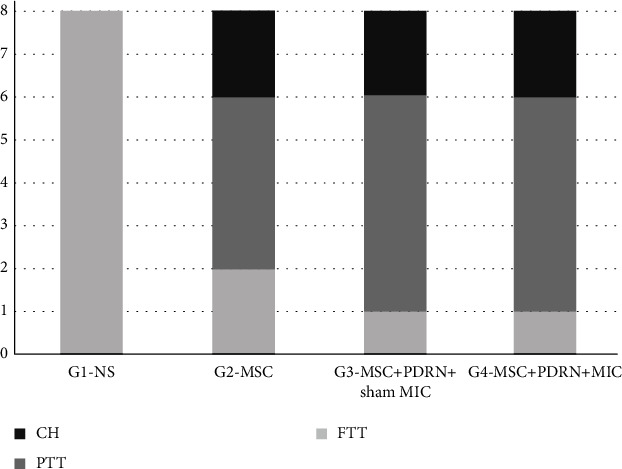
Gross morphology of tear site at 4 weeks after treatment. In G1-NS, FTRCTT were found in all 8 rabbits. In G2-MSC, FTT, PTT, and CH were observed in 2, 4, and 2 rabbits, respectively. In G3-MSC+PDRN+sham MIC, FTT, PTT, and CH were observed in 1, 5, and 2 rabbits. In G4-MSC+PDRN+MIC, FTT, PTT, and CH were observed in 1, 4, and 3 rabbits. CH: nearly complete healing (regeneration of tendon > 80%); FTT: full-thickness tendon tear; PTT: partial-thickness tendon tear.

Gross morphologic changes were evaluated before and 4 weeks after the treatment in all of the rabbits. The tendon tears were identified as either partial-thickness tendon tear or full-thickness (Figure 3). A ruler was positioned close to the center of each tear site to measure the size, and photographs of the sites were taken. Once the photographs taken were uploaded into ImageJ program (NIH, Bethesda, USA), outlines of the tear edges were traced [28]. Each supraspinatus tendon tear healing was classified as nearly complete healing (regeneration of tendon > 80%), partial healing (5% < regeneration of tendon < 80%), and no healing (regeneration < 5%) (Figure 4) [29].

### 2.5. Tissue Preparation for Histologic Examination

After euthanasia, the supraspinatus tendon areas were segmented and formalin-fixed using 10% neutral buffered formalin for a day. Paraffin embedding (Paraplast, Oxford, USA) was processed for each sample, followed by being serially sliced to 5 *μ*m thick sections.

### 2.6. Immunohistochemistry

**Figure 5:**
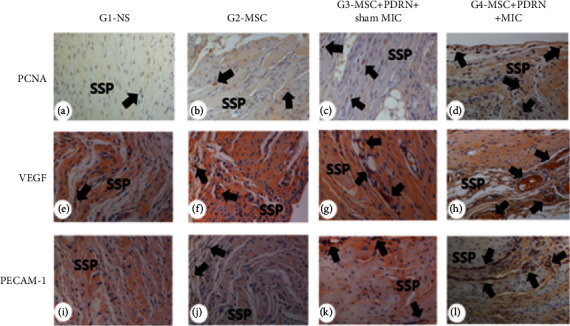
Histological findings of the supraspinatus tendon. Numerous, PCNA-stained, proliferating cell cells (black arrow, ×100) were found in regenerated tendon fibers in G4-MSC+PDRN+MIC. However, PCNA-stained cells were found less in G2-MSC and G3-MSC+PDRN, and a few PCNA-stained cells were found in G1-NS (a–d). Numerous VEGF-stained cells (e–h) and PECAM-- stained microvascular angiogenesis densities (i–l) (black arrow, ×100) were found in G4-MSC+PDRN+MIC. In G4-MSC+PDRN+MIC, VEGF-stained cells and PECAM-1-stained densities were significantly greater than those in G3-SC+PDRN. NS: Normal saline; PCNA: proliferating cell nuclear antigen; VEGF: vascular endothelial growth factor; PECAM-1: platelet endothelial cell adhesion molecule.

Each section of FTRCTTs sliced was immunohistochemically stained with mouse anti-PCNA monoclonal antibody (PC10, Santa Cruz Biotech., USA) as a proliferating cell marker (Figures 5(a)–5(d)), anti-VEGF polyclonal antibody (A-20) (Figures 5(e)–5(h)), and anti-PECAM-1 polyclonal antibody (M-20, both Santa Cruz Biotech., USA) as angiogenic markers (Figures 5(i)–5(l)) [29]. After being desiccated, paraffin-embedded sections were bathed in phosphate-buffered saline (PBS). Using citrate buffer (pH 6.0), antigen retrieval was carried out at 95°C for 30 minutes, and the sections were cooled down. To inhibit endogenous peroxidases, preincubation of the sections was performed using 0.3% hydrogen peroxide in PBS for 30 minutes, and nonspecific protein binding was blocked with 10% normal horse serum in PBS for 30 minutes [29]. At room temperature for 2 hours, primary antibodies (1 : 100-1 : 200) were used for incubation of the sections that were, then, triple washed with PBS. One selected between secondary antibody (1 : 100), biotinylated anti-mouse IgG, anti-rabbit IgG, and anti-goat IgG (Vector Laboratories) [29] was positioned on the sections and incubated at room temperature for one hour followed by triple-washing with PBS. Then, avidin-biotin-peroxidase complex (Vector Laboratories) was processed to the sections for 1 hour. Once being triple washed with PBS, the sections were treated with a peroxidase reaction with 0.05 M Tris-HCl (pH 7.6) containing 0.01% hydrogen peroxide and 0.05% 3,3′-diaminobenzidine (Sigma-Aldrich). Then, counterstaining with hematoxylin and mounting were successively performed on the sections. Using an Axiophot Photomicroscope (Carl Zeiss, Germany) interfaced with an AxioCam MRc5 (Carl Zeiss, Germany), the slides with a counterstain were examined. The intensity of positive immunostaining was evaluated [29].

### 2.7. Evaluation of Immunohistochemical Staining

**Figure 6:**
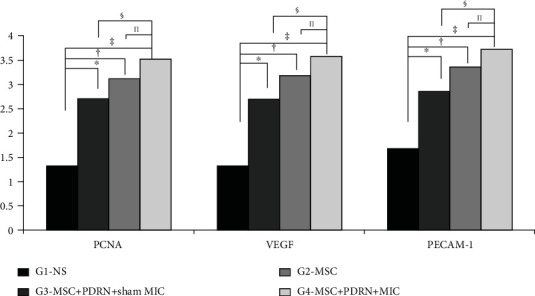
Semiquantitative scoring of histological findings based on activity of immune-stain. The proportion of cells positive for PCNA, VEGF, and PECAM-1, respectively, were scored. ^∗^*p* < 0.05, one-way analysis of variance and post hoc Tukey's test among groups.

In each group, 30 fields were chosen randomly and captured using an AxioCam MRc5 (Carl Zeiss, Germany) mounted on the Axiophot Photomicroscope. Then, they were analyzed using AxioVision SE64 (Carl Zeiss, Germany). According to a semiquantitative scoring system, PCNA, VEGF, and PECAM-1 were evaluated on the basis of the intensity and extent of staining area [29–31]. The proportion of positively stained cells was scored between 0 and 4 points. Of the points, *zero* refers to *no cells stained positive*, *one* to 1-10% positive cells, *two* to 11-33% positive cells, *three* to 34-66% positive cells, and *four* points to 67-100% positive cells (Figure 6) [29].

### 2.8. Motion Analysis

**Figure 7:**
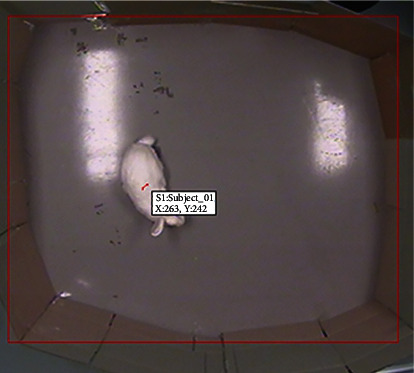
Representative image of motion analysis.

Motion analysis was carried out among the rabbits at 4 weeks post-treatment (Figure 7). The rabbits were given 30 minutes to be habituated to the open field before motion analysis began. During motion analysis, the rabbits were released in a 3 × 3 m arena to move around without restriction for 5 minutes. For movement assessment, their horizontal activities were recorded using a camera equipped with video-tracking system (SMART 3.0, Panlab, Spain). Evaluation items included walking distance, fast walking time, and average walking speed [29].

### 2.9. Statistical Analysis

Using SPSS for Windows (version 22.0, IBM Corp., USA), all statistical analyses were conducted, with *p* < 0.05 being considered significant. A chi-square test was used to compare changes of tendon tear at 4 weeks posttreatment among the 4 groups. Along with standard descriptive statistical calculations, the Kruskal-Wallis test with Bonferroni correction was performed to assess intra- and intergroup differences of gross tear size and each parameter of motion analysis. ANOVA was used to assess intra- and intergroup differences of immunohistochemical staining findings. In case statistical significance observed among groups through ANOVA analysis, Tukey's test was additionally implemented. Mean values were calculated with 95% confidence intervals.

## 3. Results

### 3.1. Gross Morphology

In G1-NS, at 4 weeks after the treatment, all of the eight rabbits (100%) were found to have a full-thickness tear (FTT). In G2-MSC, two (25.0%), four (50.0%), and two (25.0%) rabbits were found to have a FTT, a partial thickness tear (PTT), and a nearly complete healing (CH), respectively. In G3-MSC+PDRN+sham MIC, one (12.5%), five (62.5%), and two (25.0%) rabbits were found to have FTT, PTT, and CH, respectively. In G4-MSC+PDRN+MIC, one (12.5%), four (50.0%), and three (37.5%) rabbits were found to have FTT, PTT, and CH, respectively (Figure 4). Significant differences in gross morphology between the baseline and post-treatment were found in group 4, unlike in the other three groups (*p* = 0.01, Table 1).

The average tear size in gross morphology was measured as 13.79 mm^2^ in G1-NS, 3.87 mm^2^ in G2-MSC, 3.82 mm^2^ in G3-MSC+PDRN+sham MIC, and 3.68 mm^2^ in G4-MSC+PDRN+MIC. The average tendon tear size in G2-MSC, G3-MSC+PDRN+sham MIC, and G4-MSC+PDRN+MIC was significantly smaller than that in G1-NS (*p* < 0.05, Table 1).

### 3.2. Immunohistochemistry

A large number of PCNA-stained cells were detected in collagen fibers regenerated in G2-MSC, G3-MSC+PDRN+sham MIC, and G4-MSC+PDRN+MIC (Figures 5(a)–5(d)). There were significant differences in PCNA stain intensities between G2-MSC and G3-MSC+PDRN+sham MIC and between G3-MSC+PDRN+sham MIC and G4-MSC+PDRN+MIC (Figure 6).

Many VEGF-positive cells were revealed through immunohistochemistry staining (Figures 5(e)–5(h)), and PECAM-1-stained microvascular densities were identified in G2-MSC, G3-MSC+PDRN+sham MIC, and G4-MSC+PDRN+MIC (Figures 5(i)–5(l)) Also, significant differences were found in VEGF and PECAM-1 stain intensities between G2-MSC and G3-MSC+PDRN+sham MIC (*p* < 0.05, Figure 6, Table 1) and between G3-MSC+PDRN+sham MIC and G4-MSC+PDRN+MIC (*p* < 0.05, Figure 6, Table 1). In G4-MSC+PDRN+MIC, consequently, it was found that PCNA-, VEGF-, and PECAM-1-stained cells were significantly greater than those in G3-MSC+PDRN+sham MIC (*p* < 0.05, Figure 6, Table 1).

### 3.3. Motion Analyses

The results of motion analyses showed that walking distance, fast walking time, and average walking speed in the G3-MSC+PDRN+sham MIC and G4-MSC+PDRN+MIC were significantly greater than in G1-NS (*p* < 0.05, Table 1). In G4-MSC+PDRN+MIC, walking distance and mean walking speed had significantly greater values than in G2-MSC (*p* < 0.05, Table 1).

## 4. Discussion

Given the additional chemical and physical factors input, in our study, it was hypothesized that coinjection of intralesional UCB-MSCs and PDRN combined with MIC may have greater effect on regeneration of tendon tear than the injection of UCB-MSCs alone or combined injection of UCB-MSCs and PDRN in an animal model. Indeed, it has been found that rabbits provided with the combined treatment of UCB-MSCs, PDRN, and MIC showed improved severity and decreased length of their tendon tear, with regeneration of type 1 collagen fibers and improvement of cell proliferation, angiogenesis, and walking distance than those in the groups of UCB-MSCs alone and UCB-MSCs combined with PDRN. According to our knowledge, it was firstly attempted only in our study to analyze the combined therapies of UCB-MSCs and PDRN (chemical factor) combined with MIC (physical factor) for rotator cuff tear to be regenerated. Moreover, the study results are beneficial in that large volumes of UCB-MSCs and PDRN can be manufactured with a desired level of quality maintained.

Ideally, it is anticipated that the combined treatment of PDRN and UCB-MSCs would be synergistic with regard to its effects, and the existing high-priced treatment for RCTT regeneration, i.e., stem cell-based therapy, would be replaced by PDRN.

It is known that MSCs from different sources have similar biological potential; however, compared to MSCs derived from other tissues, therapeutic potential of UCB-MSCs is much greater, which attributes to its characteristics including non-invasive collection, ability to go directly towards target tissue, low level of immunogenicity, multidirectional differentiation, and comprehensive secretion profiles [32, 33]. In general, elderly patients or patients with significant comorbidities tend to have impaired function of autologous MSCs. In this context, allogeneic UCB-MSCs may be an effective treatment option particularly for the elderly or those with multiple comorbidities [34]. As mentioned above, furthermore, UCB-MSCs are able to be manufactured in large quantities. Presently, UCB-MSCs have been used as novel medicines (composed as allogeneic UCB-MSCs and HA hydrogel) for regeneration of articular cartilage in the patients with knee osteoarthritis [35].

PDRNs as well as nucleotides and nucleosides generated by PDRN degradation may enhance cells to migrate and grow, extracellular matrix protein to be produced, and levels of inflammation to decrease [36]. For purine and pyrimidine rings possibly used by salvage pathway enzymes to be provided, PDRN may penetrate into the cell with different transport mechanisms, thereby provoking nucleic acids to be synthesized on a significant energy-saving mechanism [37]. Purine nucleotides and nucleosides may be bound to receptors of a particular type and elicit different intracellular signaling pathways. They also have mitogenic effect on fibroblasts, endothelial cells, and neuroglia [38, 39]. In addition, they synergize with a variety of growth factors [40, 41]. Considering the benefits of PDRNs including safety, low manufacturing cost, no systemic toxicity, and no antigenic properties, they have been used for treatment of the bone, cartilage, and tendon diseases in clinical settings [42].

Low cell densities in tendon tissues and poor blood supplies may contribute to lack of extracellular matrix restoration required for tendon healing. In general, conservative treatment methods that prevent anatomical reduction are used for tendon healing based on external mechanical stimulations [43, 44], which may trigger cell synthesis of extracellular matrix components and cytokines to heighten cell proliferation. In addition, PDRN is in use as injectable solution. The regenerative effect of UCB-MSCs is not known to work in a dose-dependent manner, and also, the volume increment of stem cells does not increase therapeutic effects; therefore, it is very convincing combination therapy with PDRN should be considered.

In the same manner with other electrotherapies, MIC has intensity-dependent effects. According to previous study results, low-intensity electrotherapy may promote healing of tendon or ligament damages [21, 45–48]. In a study conducted by Dunn [48], electric currents ranging from 20 to 100 *μΑ* used for a guinea pig's skin wound created for the experiment, and fibroblast growth were observed in the collagen matrix in it. Several different MIC machines were used; however, maximal levels of fibroblast-growth response were found near the cathode.

In other studies [36, 49] as well, it has been demonstrated that low frequencies would be more efficient to promote connective tissue repair by changing the cell's membrane potential. In addition, a previous study [50] suggested that application of 1–8 Hz monophasic pulsed microcurrent stimulation is the optimal condition to promote the increasing the number of human dermal fibroblasts. In our previous study [16], similarly, electrical stimulation (frequency at 8 Hz and intensity of 25 *μ*A) was applied during MIC to rotator cuff tears for 60 min daily for 4 weeks, which led to reduction of the tear sizes (G1-NS group: 45%; G2-PDRN+sham MIC: 30%) in rabbit models of FTRCTT. Our study, however, did not include evaluation of effects of treatment time on the outcome, although longer duration of treatment was known to be related with higher success rates in MIC trials using other tissue types [51]. MIC tends to run at the level as low as microampere while mimicking the electrical intensity in living tissues; therefore, as expected, there have not been adverse effects or untoward events observed [49, 52].

In this study, we used a rabbit model of a chronic traumatic RCTT after 6 weeks of trauma. All rabbits were injected 6 weeks after injury to the rotator cuff and evaluated 4 weeks after injection. Studies in the rabbit supraspinatus muscle have shown fatty degeneration beginning as early as 4 weeks, with a peak at 6 weeks and slow reversal by 12 weeks [53]. FTRCTT becomes irreparable after approximately 6 weeks as the result of excessive tendon retraction and muscle atrophy and stiffening [54]. Accordingly, we selected 6 weeks for the chronic injury.

Furthermore, motion analysis of the rabbits was conducted to directly assess improvement in functional abilities of the tendons instead of focusing on mechanical properties of the regenerated tendon [9]. Yet it has not been proven motion analysis would be surpassing mechanical testing having used more often in rotator cuff tears of animal models [49, 55]; however, motion analysis, based on data from human studies, would be used as a critical tool for the assessment of the therapeutic effects of FTRCTT treatments [56].

In our study, some limitations have been identified. First, 5 × 5 mm FTRCTTs were created at slightly proximal area to the insertion point on the right supraspinatus tendon. At this point, however, these tears were located in the tendon body instead of being at the exact site of insertion. Second, the FTRCTT models used in our study were not spontaneous degenerative models but traumatic ones. Third, no complete regeneration has been observed. It is assumed more “complete” healing of rotator cuff tears might have occurred, if the outcome measurement period was extended from 4 weeks to 8 weeks or longer. Fourth, a biomechanical examination has not been performed in the regenerative tendon. Fifth, it is well known that retracted tendon healing cannot be achieved by tendon regeneration in human. It is possible that the rabbits may have better biologic healing properties. Therefore, this can be another limitation of our study. Sixth, the experiments of our study were conducted relatively in a short period of time; therefore, long-term effects of MIC would need to be assessed for further studies. Seventh, the effects of MIC alone on FTRCTT have not been assessed. Finally, in order to achieve optimal results, effects of MIC would need to be assessed by applying various frequencies and durations (e.g., <30 min and >3 hrs).

## 5. Conclusions

Injection of UCB-MSCs alone and injection of UCB-MSCs combined with PDRN with/without MIC have shown to be effective in tendon healings demonstrated by improvement in gross morphologic, histological, and motion analysis. It appears that injection of UCB-MSCs and PDRN combined with MIC might be the most effective regenerative method in rabbit models' traumatic FTRCTT.

## Figures and Tables

**Table 1:** Gross morphology, tear size, semiquantitative score of histological findings, and motion analysis at 4 weeks after treatment.

		Groups (injection regimens)		
	G1-NS	G2-MSC	G3-MSC+PDRN+sham MIC	G4-MSC+PDRN+MIC
*Gross*				
CH/PTT/FTT	8/0/0	2/4/2^∗^	1/5/2^†§^	1/4/3^‡||¶^
Tear size	13.79 ± 0.97	3.87 ± 1.87^∗^	3.82 ± 1.00^†^	3.68 ± 1.72^‡^
*Histological score*				
PCNA	1.28 ± 0.91	2.70 ± 1.08^∗^	3.12 ± 1.00^†§^	3.52 ± 0.64^‡||¶^
VEGF	1.28 ± 0.94	2.64 ± 0.93^∗^	3.18 ± 0.79^†§^	3.58 ± 0.61^‡||¶^
PECAM-1	1.69 ± 0.93	2.86 ± 0.97^∗^	3.36 ± 0.76^†§^	3.72 ± 0.49^‡||¶^
*Motion analysis*				
Walking distance (cm)	4852.75 ± 137.27	6367.38 ± 220.55	7049.50 ± 187.78^†^	7348.75 ± 177.13^‡||^
Fast walking time (%)	5.63 ± 1.42	10.12 ± 2.09	12.38 ± 0.96^†^	14.19 ± 1.78^‡^
Mean walking speed (cm/sec)	6.30 ± 0.57	10.36 ± 2.03	14.69 ± 0.69^†^	15.37 ± 0.61^‡||^

Values are the mean ± SD. The proportion of stained cells with PCNA, VEGF, PECAM-1 was scored as 0 = no cells stained, 1 = between 1%and 10%stained positive, 2 = 11%–33%, 3 = 34%–66%, and 4 = 67%–100%. NS: normal saline; MSC: human umbilical cord blood-derived mesenchymal stem cell; PDRN: polydeoxyribonucleotides; MIC: microcurrent therapy; CH: nearly complete healing (regeneration of tendon > 80%); PTT: partial-thickness tear; FTT: full-thickness tear; PCNA: proliferating cell nuclear antigen; VEGF: vascular endothelial growth factor; PECAM-1: platelet endothelial cell adhesion molecule. ^∗^*p* < 0.05 one-way ANOVA (immunohistochemical staining findings) or Kruskal-Wallis test (gross tear size and motion analysis), Tukey's post hoc test, or Bonferroni correction between groups 1 and 2. ^†^*p* < 0.05 one-way ANOVA (immunohistochemical staining findings) or Kruskal-Wallis test (gross tear size and motion analysis), Tukey's post hoc test, or Bonferroni correction between among groups 1 and 3. ^‡^*p* < 0.05 one-way ANOVA (immunohistochemical staining findings) or Kruskal-Wallis test (gross tear size and motion analysis), Tukey's post hoc test, or Bonferroni correction between groups 1 and 4. ^§^*p* < 0.05 one-way ANOVA (immunohistochemical staining findings) or Kruskal-Wallis test (gross tear size and motion analysis), Tukey's post hoc test, or Bonferroni correction between groups 2 and 3. ^||^*p* < 0.05 one-way ANOVA (immunohistochemical staining findings) or Kruskal-Wallis test (gross tear size and motion analysis), Tukey's post hoc test or Bonferroni correction between groups 2 and 4. ^¶^*p* < 0.05 one-way ANOVA (immunohistochemical staining findings) or Kruskal-Wallis test (gross tear size and motion analysis), Tukey's post hoc test, or Bonferroni correction between groups 3 and 4.

## Data Availability

All data generated/analyzed and used to support the findings of this study are included within the article.
